# Toward Integrative Bacterial Monitoring of Metolachlor Toxicity in Groundwater

**DOI:** 10.3389/fmicb.2018.02053

**Published:** 2018-10-16

**Authors:** Gwenaël Imfeld, Ludovic Besaury, Bruno Maucourt, Stéphanie Donadello, Nicole Baran, Stéphane Vuilleumier

**Affiliations:** ^1^Laboratory of Hydrology and Geochemistry, EOST-CNRS, LHyGeS UMR 7517, Université de Strasbourg, Strasbourg, France; ^2^Génétique Moléculaire, Génomique, Microbiologie, GMGM UMR 7156, CNRS, Université de Strasbourg, Strasbourg, France; ^3^Bureau de Recherches Géologiques et Miniéres (BRGM), Orléans, France

**Keywords:** groundwater contamination, microbial ecotoxicology, chloroacetanilides, biodegradation, bacterial communities, compound-specific isotope analysis

## Abstract

Common herbicides such as metolachlor (MET), and their transformation products, are frequently detected in groundwater worldwide. Little is known about the response of groundwater bacterial communities to herbicide exposure, and its potential use for ecotoxicological assessment. The response of bacterial communities exposed to different levels of MET from the Ariège alluvial aquifer (Southwest of France) was investigated *in situ* and in laboratory experiments. Variations in both chemistry and bacterial communities were observed in groundwater, but T-RFLP analysis did not allow to uncover a pesticide-specific effect on endogenous bacterial communities. To circumvent issues of hydrogeochemical and seasonal variations *in situ*, groundwater samples from two monitoring wells of the Ariège aquifer with contrasting records of pesticide contamination were exposed to different levels of MET in laboratory experiments. The standard Microtox^®^ acute toxicity assay did not indicate toxic effects of MET, even at 5 mg L^-1^ (i.e., 1000-fold higher than in contaminated groundwater). Analysis of MET transformation products and compound-specific isotope analysis (CSIA) in laboratory experiments demonstrated MET biodegradation but did not correlate with MET exposure. High-throughput sequencing analysis (Illumina MiSeq) of bacterial communities based on amplicons of the 16S rRNA gene revealed that bacterial community differed mainly by groundwater origin rather than by its response to MET exposure. OTUs correlating with MET addition ranged between 0.4 to 3.6% of the total. Predictive analysis of bacterial functions impacted by pesticides using PICRUSt suggested only minor changes in bacterial functions with increasing MET exposure. Taken together, results highlight MET biodegradation in groundwater, and the potential use of bacterial communities as sensitive indicators of herbicide contamination in aquifers. Although detected effects of MET on groundwater bacterial communities were modest, this study illustrates the potential of integrating DNA- and isotopic analysis-based approaches to improve ecotoxicological assessment of pesticide-contaminated aquifers.

**GRAPHICAL ABSTRACT:**
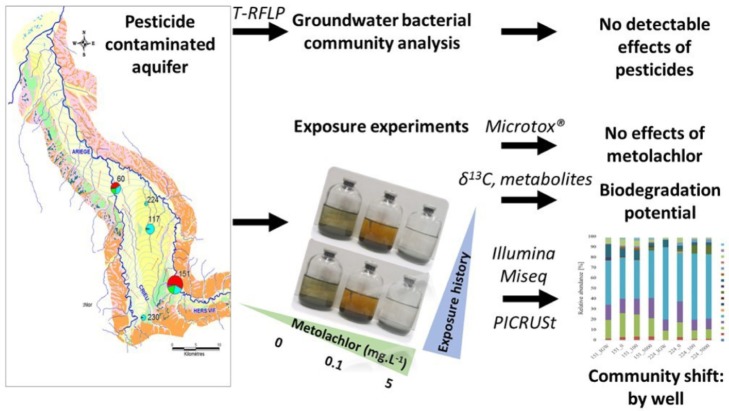
An integrative approach was develop to investigate *in situ* and in laboratory experiments the response of bacterial communities exposed to different levels of MET from the Ariége alluvial aquifer (Southwest of France).

An integrative approach was develop to investigate *in situ* and in laboratory experiments the response of bacterial communities exposed to different levels of MET from the Ariége alluvial aquifer (Southwest of France).

## Introduction

Ongoing intensive use of pesticides leads to accumulation of pesticide mixtures and their transformation products (TP) in groundwater ecosystems (Postigo and Barcelo, 2015). While pressure on groundwater resources is on the increase, the extent of ecosystem disturbance following exposure to pesticides remains difficult to evaluate precisely. In this context, sensitive monitoring approaches hold potential to address key ecological questions, such as the response of groundwater ecosystems to punctual and chronic pesticide exposure, in terms of both taxonomic and functional alterations (Imfeld and Vuilleumier, 2012). The microbial compartment, in particular, may be accessed for this purpose and today, with unprecedented sensitivity through high-throughput sequencing.

The response of microbial communities to pesticide exposure has often been addressed for the soil compartment (Imfeld and Vuilleumier, 2012; Jacobsen and Hjelmso, 2014; Ju et al., 2017). For groundwater systems, in contrast, focus has been mainly limited to evaluation of pesticide dissipation (Tuxen et al., 2002; de Lipthay et al., 2003; Liebich et al., 2009; Caracciolo et al., 2010), with only a few studies so far addressing alterations of bacterial communities following exposure, and often conflicting observations. For instance, profiles of carbon substrate usage were altered by elevated levels of nitrate (>15 mg L^-1^) and herbicides (>0.03 μg L^-1^) in an oxic aquifer (Janniche et al., 2012), while in contrast, alterations of groundwater bacterial community following herbicide exposure (<50 μg L^-1^) were not detected (de Lipthay et al., 2004). More recently, SSCP fingerprinting and monitoring of functional genes for triazine degradation and nitrate usage suggested that bacterial composition was affected by the now banned triazines in historically contaminated groundwater (Mauffret et al., 2017), but not by currently used chloroacetanilide herbicides.

Chloroacetanilide pesticides are used for control of annual weeds, mainly on corn, sugar beet and sunflower, and belong to the top 10 pesticide classes in current use worldwide (Food and Agriculture Organization of the United Nations (FAO), 2013). They are frequently detected in groundwater together with their transformation products (TP), notably ethane sulfonic and oxanilic acids (Kalkhoff et al., 2012; Baran and Gourcy, 2013; Sidoli et al., 2016). Metolachlor (MET) is a chloroacetanilide herbicide in massive used worldwide, and one of the top-five of pesticides detected in France (Lopez et al., 2015) and in the EU (Loos et al., 2010). MET was brought to the market in 1976 as a racemic compound (*rac*-metolachlor). It was subsequently replaced in the 2000s by *S*-MET, which is enriched (approximately 86%) in the herbicidally more active 1′*S*-enantiomer (Ma et al., 2006; Xu et al., 2010). Although biodegradation of MET has been reported (Singh and Singh, 2016), enzymes and pathways for microbial transformation of MET are still unknown, thus preventing specific monitoring of microbial MET degradation.

In this context, the purpose of this study was to evaluate the response of groundwater bacterial community to exposure to MET and its transformation products, as well as MET degradation potential. Issues of field hydrogeochemical heterogeneity in the response of bacterial community in the Ariège alluvial plain (Southwest France) were circumvented by laboratory microcosm experiments of MET exposure. Groundwater samples from two monitoring wells with contrasting records of pesticide contamination were exposed to different levels of MET *in labo*. MET biodegradation was evaluated based on dissipation and patterns of transformation products, as well as on change of carbon isotope composition using compound-specific isotope analysis (CSIA), and the bacterial community response was evaluated by 16S rRNA amplicon sequencing.

## Materials and Methods

### Chemicals

Racemic metolachlor (C_15_H_22_ClNO_2_, (S)-2-Chloro-N-(2-ethyl-6-methyl-phenyl)-N-(1-methoxypropan-2-yl)acetamide) was purchased from Sigma-Aldrich (>99% purity). Stock solutions for spiking were prepared at 1 g L^-1^ in acetonitrile (ACN).

### Groundwater Site

**FIGURE 1:**
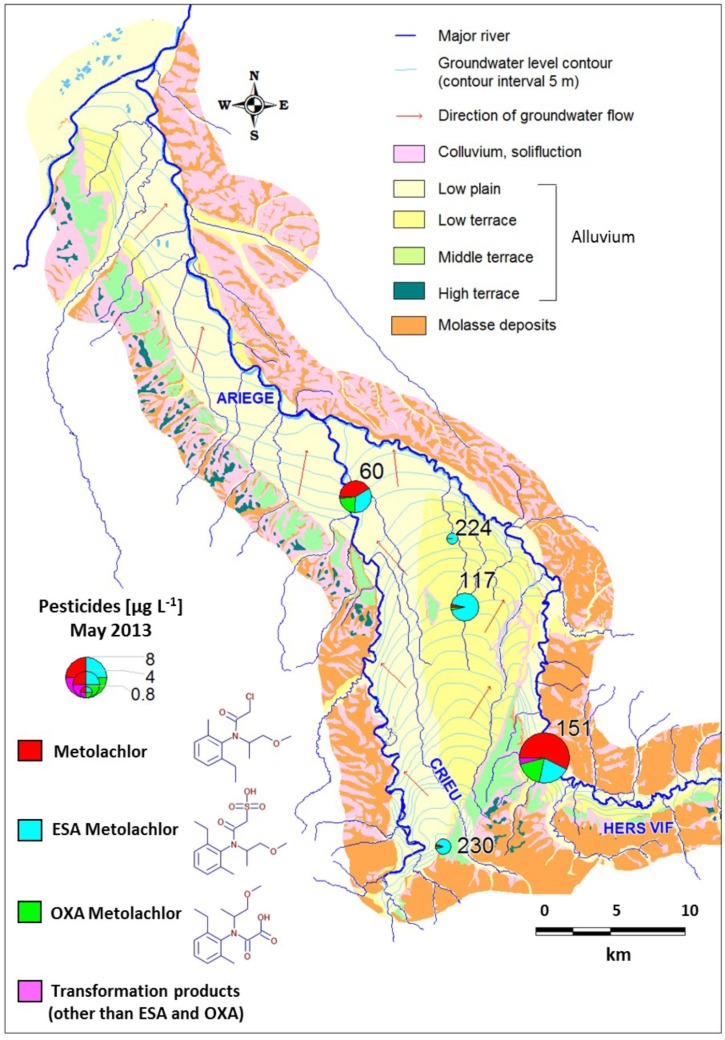
Site map, location of the wells and metolachlor concentrations in the Ariège alluvial plain (May 2013).

The study area (538 km^2^) covers the alluvial domain of the Ariège river (**Figure 1**). The alluvial plain lies above Aquitanian (Miocene) and Stampian (Oligocene) molasse deposits. Alluvium transported by the Ariège deposited as silt on the molasse, in five terrace levels with similar physicochemical composition. Terrace levels differ in their degree of pebble weathering and pedological evolution. The sand-and-gravel alluvium of the lower terrace and the lower plain defined a continuous unconfined aquifer feeding rivers Ariège and Hers-Vif. The thickness of the unsaturated zone varies between a few meters and up to 10 m locally. The study area features mostly cultivated farmland, including corn.

### Groundwater Collection

Groundwater sampling was conducted in May, July, September and December 2012, and February and May 2013. The five sampled monitoring wells 151, 60, 117, 230, 224 (**Figure 1**) define a decreasing concentration gradient of MET and its transformation products (TP), and present contrasted hydrochemical composition (**Supplementary Section C**). Samples were collected after pumping and discarding three purge volumes, to ensure pH and electrical conductivity stabilization. Temperature, pH, electrical conductivity (EC, standardized to 25°C), redox potential (Eh) and dissolved oxygen (O_2_) were measured on-site using a WTW multi 340i meter equipped with a Sentrix 81 WTW pH electrode and a Tetra Con 325 WTW EC electrode (Weilheim, Germany). For analyses of anions and major cations, samples were separately collected in 100 mL PE bottles after filtration through a 0.45 μm PVDF filter. Samples for major cations analyses were acidified to pH 2 with ultrapure HNO_3_. For MET and TP analysis, groundwater (1 L) was collected in a glass bottle. All samples were placed on ice for transportation to the laboratory and stored at 4°C until chemical analysis.

### Laboratory Exposure Experiments

Groundwater samples (10 L) from monitoring wells 151 and 224 with contrasting records of contamination (**Supplementary Section D**) were collected in February 2014. Well 151 has a history of chronical exposure to MET (5.1 ± 4.0 μg L^-1^, mean ± σ, *n* = 6 May 2012 – May 2013). In contrast, MET had not been detected in well 224 (**Supplementary Section D**). Hydrochemical and bacterial composition of initial groundwater used for preparing the exposure experiments was analyzed for comparison with spiked and incubated groundwater.

To compare the response of bacterial communities exposed to MET, groundwater subsamples (experimental repetitions) originating from initial 10 L groundwater samples collected in each well, were incubated *in labo* in 1 L glass vials. Each vial contained 700 mL of groundwater. Three exposition doses were compared for each of the two selected wells: background MET concentrations (no MET addition), and added MET at either 0.1 mg L^-1^ (equivalent to severe chronic contamination) or 5 mg L^-1^ (equivalent to punctual source contamination). An experimental repetition (i.e., two vials) was prepared from each of the two selected wells and for each of the three exposition doses.

The MET standard stock solution (1 g L^-1^ in can) was dissolved in distilled water, and ACN was evaporated after 6 h stirring under a fume hood. MET aqueous solutions were filter-sterilized through a 0.2 μm syringe filter (Rotilabo^®^, Carl Roth^®^, France) before spiking. To ensure initial homogeneisation following spiking with MET, laboratory microcosms were shaken at 100 rpm for 1 h before incubation at the same temperature of 20°C. In experiments without MET addition, the same procedure was followed using pure ACN evaporated in distilled water to account for possible effects of ACN traces. ACN was never detected in the headspace of MET aqueous solutions (data not shown). Control experiments (performed in duplicate) consisted in filter-sterilized water spiked with MET to evaluate abiotic MET dissipation. To maintain aerobic conditions while limiting water loss and avoiding contamination, a 0.22 μm syringe filter was mounted on a syringe tip stuck through the vial cap.

Oxygen concentration in each microcosm was monitored weekly with oxygen sensor spots (PreSens Precision Sensing GmbH, Germany) fixed to the inner face of the bottles before autoclaving. Oxygen concentrations from 5 to 8 mg L^-1^ confirmed oxic conditions throughout the experiment (**Supplementary Section C**). Laboratory experiments with or without addition of MET were incubated for 21 days, a duration typical of reported MET half-life values (The Pesticide Properties DataBase [PPDB], 2006). Mean groundwater temperature was 15 ± 1.3°C. Experiments were incubated at the reference temperature for standardized testing of pesticides based on OECD guidelines of 20°C (Organisation for Economic Co-operation and Development [OECD], 1981). Experiments were incubated without stirring because (i) oxygen was continuously monitored and was not rate-limiting for bacterial growth (Hensler and Schedel, 1991), and (ii) stirring may artefactually alter the diversity of groundwater bacterial communities adapted to heterogeneous aquifer microenvironments and non-turbulent flows.

At the end of incubations, water samples from each duplicate experiment were collected using sterile glass syringes for MET, TP analysis (2 × 15 mL) and the Microtox^®^ assay (2 × 15 mL) (see below). Remaining groundwater of each repetition experiment (2 × 670 mL) was filtered through a unique sterile 0.2 μm cellulose membrane (Millipore, Billerica, MA, United States) to obtain DNA in sufficient and representative quantities for Illumina sequencing (Aird et al., 2011). Each membrane was stored at -20°C in sterile 50 mL plastic Falcon tubes until further processing.

### Chemical Analysis

#### Hydrogeochemical Analysis

Groundwater samples were analyzed at BRGM Orleans using ICP-AES (Ca^2+^, Na^+^, K^+^, Mg^2+^ with 5% uncertainty), ion chromatography (Cl^-^, SO_4_^2-^, NO_3_^-^ with 10% uncertainty) and potentiometric methods according to N EN ISO 9963-1 (HCO_3_^-^, CO_3_^2-^ with 5% uncertainty). Total organic carbon (TOC) and dissolved organic carbon (DOC) were quantified according to NF EN 1484 (1997) procedures.

#### Pesticide and TP Analysis of Field Samples

Pesticides in groundwater, including MET and its neutral TP, were extracted using a Gilson GX 274 ASPEC solid phase extraction (SPE) system, with Oasis HLB (6 mL - 500 mg) cartridges (Waters) and quantified as described elsewhere (Amalric et al., 2013). Briefly, cartridges were successively conditioned at pH 7 with 5 mL acetonitrile (ACN), 5 mL methanol (MeOH), and 5 mL deionized water (HPLC grade) at a flow rate of 1 mL min^-1^. Water samples (1 L) were loaded at a flow rate of 5 mL min^-1^. After drying by flushing with pure N_2_ (30 min), analytes were eluted twice successively with 4 mL ACN at a rate of 1 mL min^-1^. Sample extracts were concentrated down to 1 mL under a gentle stream of nitrogen at ambient temperature. For extraction of ionic compounds, cartridges were conditioned at a flow rate of 1 mL min^-1^ with MeOH (5 mL), followed by ACN (5 mL) with 0.2% v/v acetic acid. The water sample (1 L; pH 6–8), was loaded at a flow rate of 5 mL min^-1^. After 30 min of drying, analytes were eluted twice successively with MeOH (4 mL) at 1 mL min^-1^, and eluates concentrated to 1 mL as described above.

Quantification of pesticides and TP was carried out by a Waters Ultra Performance Liquid Chromatography (UPLC) system coupled to a Waters micromass MSMS (Waters Quattro-Premier XE/Q). Chromatographic separation for neutral and ionic compounds was achieved with a Waters Acquity UPLC BEH C18 column (2.1 mm × 150 mm, particle size 1.7 μm). The mobile phase consisted of a gradient of (A) water/0.05% formic acid and (B) acetonitrile/0.05% formic acid for neutral molecules, and (A) water/0.007% formic acid and (B) methanol/0.007% formic acid for ionic molecules, established at a flow rate of 0.4 mL min^-1^. Limits of quantification were 5 ng L^-1^ for MET, 10 ng L^-1^ for MESA and MOXA, and between 5 and 20 ng L^-1^ for neutral TP.

#### Carbon Stable Isotope Analysis of MET

The carbon isotope composition of MET in the laboratory exposure experiment was analyzed by adapting a previously described protocol (Elsayed et al., 2014) (see **Supplementary Section A** for the detailed protocol). Briefly, the GC-C-IRMS system consisted of a TRACE^TM^ Ultra Gas Chromatograph (Thermo Fisher Scientific) coupled to an isotope ratio mass spectrometer (DeltaV Plus, Thermo Fisher Scientific) via a GC IsoLink/Conflow IV interface. Reproducibility of triplicate measurements was ≤0.2‰ (1 σ). Carbon isotope ratios were reported in δ notation in parts per thousand [‰], relative to the international carbon isotope standard Vienna Pee Dee Belemnite (V-PDB).

### Enumeration of Viable Cells and Microtox^®^ Assay

Enumeration of total viable bacteria was carried out by plating out on R2A agar (typically used for drinking water) after incubation at 20°C for 48 h. Toxicity of MET in laboratory exposure experiments was assessed by the standard Microtox^®^ test (HACH Lange, Düsseldorf, Germany) following a standard protocol (Jennings et al., 2001). All materials for analysis (test strain and reagents) were supplied in the commercial kit, and luminescence was measured in Nunc 96-well white polystyrene microtiter plates (Thermo Scientific) using a Luminoskan Ascent luminometer (Thermo Scientific).

### Biomolecular Analyses

#### DNA Extraction

Total DNA was extracted from filters with the PowerWater^®^ DNA Isolation Kit (MO BIO, Carlsbad, CA, United States) following manufacturer’s instructions. Concentrations of DNA were determined using the Quant-it PicoGreen dsDNA Assay Kit (Invitrogen, Carlsbad, CA, United States).

#### T-RFLP Analysis of Groundwater Samples

Bacterial 16S rRNA gene fragments (0.9 kb) were PCR-amplified using 5-carboxyfluorescein (6-FAM) labeled 27 f and 927 r primers as described previously (Mauffrey et al., 2017). T-RFLP electrophoregrams were analyzed with PeakScanner V1.0 (Applied Biosystems, Carlsbad, CA, United States). Noise cancelation, peak alignment and matrix (samples × T-RFs) generation was performed using T-REX^1^ (Culman et al., 2009). Peak heights were normalized to the same total fluorescence per sample, and resulting data matrices were used for statistical analysis.

#### Illumina MiSeq Sequencing and Data Processing

Sequencing of laboratory exposure experiment samples was performed at INRA-UR0050-LBE (Narbonne, France) using Illumina MiSeq. The 16S rRNA gene spanning hypervariable region V4 and V5 was amplified in a two-step process including a set of multiplex indexed primers (U515F 5′-GTGYCAGCMGCCGCGGTA-3′ and U909R 5′-CCCCGYCAATTCMTTTRAGT-3′) (Walters et al., 2011). Individual PCR products were purified and quantified using Qubit dsDNA HS Assay Kit^®^ (Invitrogen, France), and a pool of equimolar amounts of each amplicon was prepared. A final gel purification step ensured elimination of non-specific products. The combined library was loaded onto the Illumina MiSeq Platform using a standard MiSeq paired end (2 × 250 bp) flow cell and reagent cartridge. Denoising, chimera checking, generation of operating taxonomic units (OTUs) and taxonomic classification were performed using Mothur software package v.1.33.2 (Schloss et al., 2009) by following the default parameters from the analysis pipeline of MiSeq SOP^2^. The detailed protocol is provided in **Supplementary Section B**. Obtained sequences were deposited in the NCBI BioProject database (BioProject ID: PRJNA393085).

Obtained sequences analyzed using Mothur were clustered to define OTUs at 98% sequence identity. A subsample of sequences was randomly selected to obtain equally sized datasets according to the standard operating procedure (Schloss et al., 2009). The resulting datasets were used to calculate Shannon and inverse Simpson diversity indices and the Chao1 richness estimate using R, and for rarefaction analysis as previously described (Babcsányi et al., 2017).

### Data Analysis

#### PICRUSt Analysis

Functional profiling of bacterial communities was predicted by Phylogenetic Investigation of Communities by Reconstruction of Unobserved States (PICRUSt) (Langille et al., 2013) by classifying bacterial sequences against the Greengenes database (McDonald et al., 2012); 13 August 2013 version. OTUs were defined based on automatic 98% sequence identity. The biom file generated within Mothur command-line was uploaded into Galaxy^3^ for pre-processing, the output file was analyzed using STAMP (Parks et al., 2014), and retrieved metagenomic profiles were further analyzed using multivariate statistical analysis.

#### Statistical Analysis

Multivariate analyses were carried out within R Development (R Core Team, 2017). To visualize dissimilarities in groundwater bacterial community structures obtained from T-RFLP analysis, non-metric multidimensional scaling (NMDS) based on Bray-Curtis dissimilarities of Hellinger-transformed data was performed. The relationship between the 27 groundwater community profiles and the 30 physicochemical variables (12 physicochemical variables and 18 pesticides and their TP; see **Supplementary Sections C,D**) was investigated by fitting environmental vectors *a posteriori* onto the NMDS, and their significance was assessed with a Monte-Carlo permutation test (1000 permutation steps). Analysis of similarities (ANOSIM) based on Bray–Curtis dissimilarities were used to infer statistical differences between groups of community profiles.

Illumina MiSeq sequencing data and PICRUSt profiles were classified separately by cluster analysis to investigate the relationship between bacterial communities exposed to different levels of MET. Hierarchical cluster analysis was carried out to identify robust groupings of sequence data and PICRUSt profiles based on the distances supplied in a hierarchical manner. For both analyses, distance between Hellinger-transformed datasets was computed based on the Hellinger distance. A hierarchical cluster analysis was performed on the resulting dissimilarity matrix using Ward’s method (Ward, 1963). The optimal number of clusters was determined using Spearman’s rank correlations (Becker et al., 1988).

## Results and Discussion

### Groundwater Bacterial Communities and Effect of Environmental Variables

**FIGURE 2:**
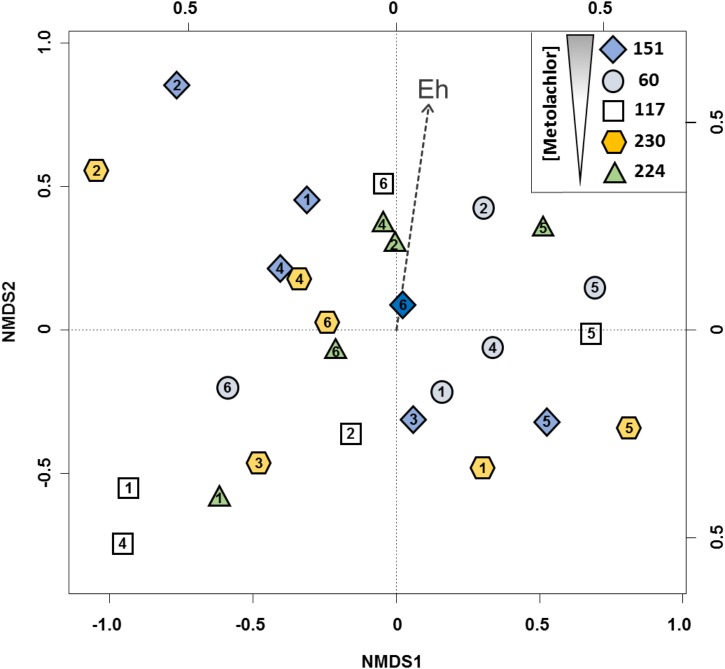
2D-NMDS ordination of bacterial community profiles from the Ariège alluvial plain groundwater obtained by T-RFLP analysis from 5 wells 151, 60, 117, 230, 224. Wells feature a decreasing gradient of MET concentrations, numbers in the objects indicate the groundwater collection campaigns. 1: May 2012, 2: July 2012, 3: September 2012, 4: December 2012, 5: February 2013, and 6: May 2013. Plot stress = 0.19.

T-RFLP fingerprints of PCR-amplified 16S rRNA gene fragments were used to follow dominant community members and compare their variation in samples collected from different origins and time points from the Ariège aquifer. Compared to DGGE, TGGE or SSCP, T-RFLP was used for its resolving power and because T-RFs can be used for cross-referencing with other studies (Hewson and Fuhrman, 2006). Strong spatial and temporal variations in bacterial communities were revealed by ordination of T-RFLP profiles (**Figure 2**). *A posteriori* fitting of physicochemical and pesticide variables revealed that changes in bacterial community composition correlated significantly only with redox potential (*p* < 0.05).

These results are consistent with previous data from a related study of the Ariège aquifer that suggested that bacterial communities were influenced by hydrogeological conditions and triazines (Mauffret et al., 2017). In this study, however, correlation with pesticide levels was not apparent. Indeed, changes in overall bacterial composition following disturbance by chronic and low exposure in multi-contaminated groundwater are unlikely to be easily detected due to often large and uncontrollable effects of changes in physico-chemical conditions *in situ*, unlike the effect of acute and punctual contamination by industrial solvents for example (Imfeld et al., 2008; Rossi et al., 2012). We focused on the currently used and often detected MET herbicide rather than on banned historical contaminants such as triazines, which continue to persist in groundwater. To test whether MET and its metabolites affected specifically bacterial assemblages, the 27 samples were clustered for comparison into two homogenous subgroups of lower (<1.4 μg L^-1^, *n* = 13) and higher (>1.4 μg L^-1^, *n* = 14) concentrations of both MET and transformation products (TP). The two clusters of bacterial assemblages did not differ (*p* < 0.01; effect size Hedges’ *g* < 0.10), suggesting little effect of MET and its TP on groundwater communities. This also supports the notion that the response of bacterial groundwater communities to MET *in situ* cannot easily be teased apart from the effects of variations of hydrogeological conditions using T-RFLP. Worthy of note, T-RFLP of 16S rRNA genes, as other fingerprinting techniques, may suffer not only from a dependence of the data obtained on the protocol used for extraction of nucleic acids from groundwater samples, which may limit reproducibility between groundwater wells, but also from the high differential required to reliably detect a dose-dependent response at environmentally relevant concentrations. Thus, an *in labo* microcosm approach under controlled conditions was subsequently used in order to characterize MET degradation potential and composition of bacterial communities from contaminated groundwater as a function of MET exposure.

### Effect of MET Exposure on Bacterial MET Degradation

Variations in physicochemical conditions and heterogeneity of aquifer structure may not only affect the composition of bacterial communities, but also its metabolic activity (Imfeld et al., 2011; Rossi et al., 2012). We thus evaluated whether different levels of MET exposure affected pesticide degradation as the key function of interest in the contaminated aquifer, and whether the bacterial response correlated with historical records of groundwater contamination.

The extent of MET dissipation in exposure experiments with groundwater from wells 151 (chronic exposure to MET) and 224 (historical records of very low MET detection) ranged from 20 to 51% after 21 days (**Table 1**). No MET dissipation was observed in the abiotic control (data not shown). Change in carbon isotope composition after 50% MET dissipation during this experiment (**Table 1**) highlight MET biodegradation, and are consistent with previous studies (Elsayed et al., 2014; Elsner and Imfeld, 2016). Specifically, MET was slightly enriched in ^13^C (>1‰) in the 5 mg L^-1^ experiments compared to initially spiked MET. Historical MET exposure also correlated with a more limited extent of MET dissipation in groundwater from well 151 exposed to 0.1 mg L^-1^ MET. Since hydrochemical conditions were the same in the different microcosms, it is likely that they do not affect degradation (**Table 1** and **Supplementary Section E**). Extent of MET degradation did not change significantly across experiments, including at high MET concentrations. This suggests a lack of community sensitivity to MET exposure levels, or alternatively, fast recovery or adaptation of groundwater functions to degrade MET at high MET exposure levels. This phenomenon was observed previously for different pesticides in soil (Jacobsen and Hjelmso, 2014).

**Table 1:** MET degradation, degradation product, carbon stable isotope composition, and bacterial toxicity in the groundwater exposure experiments (21 days of incubation at 20°C).

Well	MET addition [mg L^-1^]	MET [mg L^-1^]	Transformation products (TP) [μg L^-1^]^b^					MET degradation		Toxicity^e^
					
			Total	OXA	ESA	Main TP	Other than OXA and ESA	Extent [%]	Δδ^13^C^d^	Luminescence [%]
151	0	0.005^a^	6.0	2.9	3.0	ESA	0.1	<LOQ	<LOQ	105 ± 14
	0.1	0.081^b^	7.2	3.1	3.4	ESA	0.7	20	<LOQ	101 ± 9
	5	2.450^b^	25.2	2.3	2.1	MET morpholinone^c^	20.8	51	1.4 ± 0.5	103 ± 12
224	0	<LOQ^a^	0.9	<LOQ	0.9	ESA	<LOQ	<LOQ	<LOQ	101 ± 5
	0.1	0.054^b^	1.4	<LOQ	0.9	ESA	0.5	46	<LOQ	102 ± 3
	5	2.456^b^	30.6	<LOQ	0.6	MET morpholinone^c^	30	51	1.2 ± 0.5	102 ± 8


*n.d., not detected; n.a., not assessed.*

*^*a*^MET concentration in collected groundwater.*

*^*b*^MET and TP concentrations measured after 21 days of groundwater incubation at 20°C.*

*^c^4-(2-ethyl-6-methylphenyl)-5-methyl-3-morpholinone.*

*^*d*^The error given for Δδ^*13*^C values was calculated from ≥3 measurements for each sample, via error propagation based on ± one standard deviation of mean δ^*13*^C-values.*

*^*e*^The error given for luminescence (%) values corresponds to ± one standard deviation of the mean from ≥3 measurements for each sample.*

### Effect of Groundwater on Production of MET Transformation Products

The spectrum of known polar MET transformation products (TP) was analyzed to evaluate production and dissipation of MET TP (Amalric et al., 2013; Sidoli et al., 2016) in laboratory experiments. Both MET OXA and ESA were detected in experiments with groundwater from well 151, whereas only ESA was detected in those with groundwater from well 224 (**Table 1**). This corresponded to TP patterns in the initial groundwater wells. ESA and OXA formation was suggested to be mediated by glutathione-S-transferases (GSTs) (Graham et al., 1999), but no enzyme acting on chloroacetanilides has yet been described. The previously reported abiotic dechlorination of chloroacetanilides to ESA by reduced sulfur species under sulfate-reducing conditions (Cai et al., 2007; Bai et al., 2013) is unlikely in this case because of the aerobic conditions in our experiments. Thus, OXA could be preferentially degraded in experiments with well 224 groundwater, although OXA was not detected in initial groundwater (**Supplementary Section D**). MET may also be degraded to ESA and OXA *via* other yet unknown enzymatic pathways (Barbash et al., 1999). Nevertheless, TP other than MET OXA and ESA were often prominent, most notably in experiments spiked with 5 mg L^-1^ MET (**Table 1**). The detected TP suggested that degradation pathways involving 4-(2-ethyl-6-methylphenyl)-5-methyl-3-morpholinone, a major photolysis product (Mathew and Khan, 1996), or hydroxymetolachlor, an important fungal and bacterial degradate (Sanyal et al., 2000), were operative in our microcosms and thus potentially also in groundwater. Alternative degradation pathways that do not involve OXA and ESA as intermediates were previously observed in artificial wetlands (Elsayed et al., 2015) and at the agricultural catchment scale (Marie et al., 2017).

Worthy of note, the TP/MET ratio was highest (about 9%) in the experiment with 151 groundwater (chronical MET exposure) at 0.1 mg L^-1^ MET, but remained below 3% in the other experiments. This suggests that TP were rapidly degraded, since accumulation of TP resulted in apparent reduction of MET dissipation rates in some experiments (**Table 2**). Alternatively, it is possible that other unknown but relevant TP of MET were not detected.

**Table 2:** Richness, diversity and distribution of bacterial OTUs at 98% sequence identity, and relative distribution of abundant and rare OTUs in the initial groundwater and the groundwater exposure experiments (21 days of incubation at 20°C).

Well	Exposure to MET [mg L^-1^]	Chao 1 (*S*_Chao1_) Richness	Shannon (*H′*) diversity	Simpson (*S*) diversity (inv.)	Abundance range [%]			
					
					>10	<10-1	<1-0.1	<0.1
151	Initial groundwater	8703	3.6	12	22.9	42.6	15.0	19.5
	0 (no MET addition)	6900	4.6	30	11.0	50.3	14.5	24.3
	0.1	7447	4.9	47	0	58.8	14.1	27.1
	5	6829	4.6	32	10.7	46.8	12.4	30.1
224	Initial groundwater	7589	3.6	13	22.6	55.9	9.1	12.4
	0 (no MET addition)	8318	4.1	23	21.3	51.7	9.5	17.5
	0.1	7650	3.9	15	19.9	55.1	9.2	15.8
	5	6741	3.9	16	18.9	54.7	10.6	15.9


### Potential Toxicity Effects of MET Exposure on Groundwater Bacterial Communities

Viable cell counts on solid medium were very similar in all microcosms experiments irrespectively of MET exposure level (**Supplementary Section F**), suggesting that toxic effects of MET on the bacterial compartment were minor. Potential effects of MET and its degradates produced in microcosms on bacterial metabolic activity were further probed using the standard Microtox^®^ bacterial assay, which relies on disruption of luminescence production by a tester bacterial strain. The standard Microtox^®^ test was chosen as it is routinely used, standardized and cost-effective. Previously reported EC_50_ values ranging from 15.3 to 19.1 mg L^-1^ (Kock et al., 2010; Souissi et al., 2013) for pure MET are very high, and indeed, no effects of MET in pure water were detected below 30 μM (i.e., 8.5 mg L^-1^ MET in pure water), with an EC_20_ for MET higher than 40 μM (i.e., 11.4 mg L^-1^ MET in pure water). In addition, microcosm groundwater samples after 21 days of incubation did not produce detectable effects (**Supplementary Section G**). This confirms that as MET itself, transformation products of MET in microcosms, be them identified or not, did not affect metabolism of the Microtox^®^ tester strain. Altogether, our data confirm that the Microtox^®^ is of poor value for ecotoxicity assessment of MET, and that cultivation-dependent approaches may often be less sensitive than cultivation-independent approaches to detect potential bacterial toxicity in groundwater.

We thus turned to high-throughput sequencing of 16S rRNA amplicons by Illumina MiSeq to evaluate to what extent this could help characterize how the groundwater bacterial compartment responds to MET (Tan et al., 2015).

### Effect of MET Exposure on Groundwater Bacterial Community

A total of 19 545 OTUs were obtained from the eight groundwater experiments. OTUs covered 26 phyla, 179 families and 346 genera. Sequencing depth allowed to retrieve a representative portion of total bacterial diversity (**Supplementary Section H**), with diversity indices reaching asymptotes (**Supplementary Section I**), indicative of sufficient sampling depth. The effect of MET exposure on groundwater bacterial communities from the eight groundwater experiments was assessed based on (i) diversity indices, (ii) relative distribution of abundant and rare OTUs, and (iii) cluster analysis of Illumina MiSeq sequencing data to evaluate changes in bacterial composition. Overall, Shannon and inverse Simpson diversity indices, but not the S_chao1_ estimator, were higher in experiments with well 151 groundwater than with well 224 groundwater (**Table 2**). This mainly reflects the relative distribution of abundant and rare OTUs in the two wells. Over 70% of OTUs in well 224 experiments had high, greater than 1% abundance (**Table 2**). By contrast, 34–42% of OTUs in experiments with well 151 groundwater were either rare (<1% abundance) or very rare (<0.1% abundance). In the latter microcosms, very rare OTUs slightly increased with increasing addition of MET, whereas they tended to decrease in experiments with well 224 groundwater (**Table 2**). As previously reported for soils (Jacobsen and Hjelmso, 2014), however, the variations in groundwater community composition observed here were not associated with differences in MET degradation, which did not differ significantly across microcosms (**Table 2**).

**FIGURE 3:**
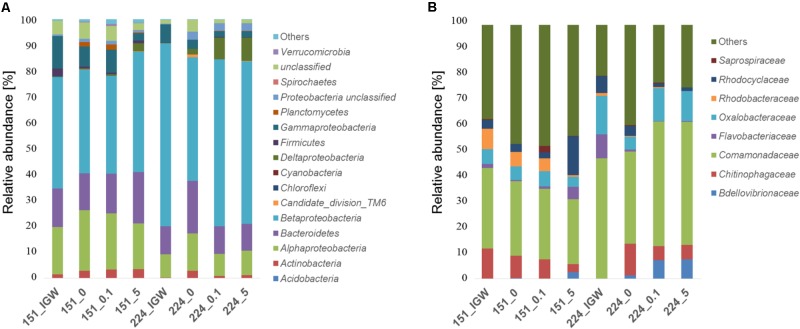
Relative abundance (%) of bacterial phyla/classes **(A)** and families **(B)** in groundwater from wells 151 and 224 (IGW: initial groundwater) and in laboratory experiments of groundwater without MET addition (0) or exposed to 0.1 mg L^-1^ and 5 mg L^-1^ MET during 21 days at 20°C.

Analyzing bacterial composition patterns in more detail, *Betaproteobacteria* (60% sequence identity clustering, phylum/class level), represented over 60% of sequences in initial groundwater and in experiments with well 224 groundwater involving MET addition, whereas they accounted for less than 45% of sequences in well 151 groundwater experiments (**Figure 3A**). In all groundwater experiments, the 8 most abundant families (80% clustering) represented 55% of sequences, with *Comamonadaceae* the most abundant (>25%) (**Figure 3B**), while at 98% clustering, the 25 most abundant OTUs made up 90% of total sequences. *Comamonadaceae* increased with MET addition in experiments with groundwater from well 224 (>45%), compared to the experiment without MET addition (<37%). *Rhodobacteraceae* became very rare (<0.1%) at 5 mg L^-1^ exposure in experiments with groundwater from both wells. Microbial diversity of MET-contaminated environments and enzymes and pathways for MET degradation, in particular in groundwater, are still unknown, preventing comparison with other studies.

**FIGURE 4:**
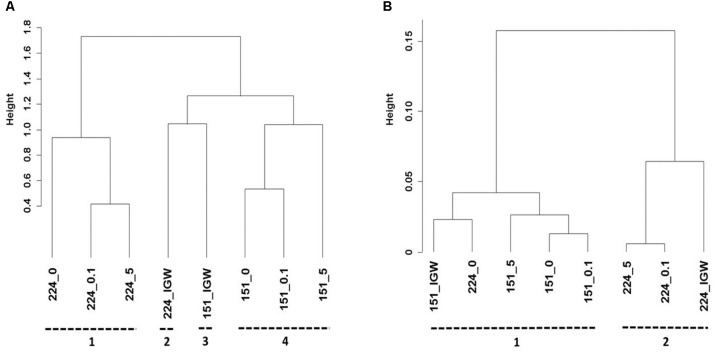
Hierarchical cluster analysis of Illumina MiSeq sequencing data **(A)**, and PICRUSt profiles **(B)** of groundwater from wells 151 and 224 (IGW: initial groundwater) without MET addition (_0) or exposed to 0.1 mg L^-1^ (_0.1) and 5 mg L^-1^ (_5) MET during 21 days at 20°C in laboratory experiments. Clusters identified using Spearman’s rank correlations are indicated by numbers.

Cluster analysis was performed to better understand the potential origin of the observed variations in bacterial composition between microcosms, revealing four distinct clusters (**Figure 4A**). Bacterial communities of the initial groundwater clearly differed from samples obtained following laboratory incubation, suggesting differentiation into distinct communities in the different experiments. Samples were mainly discriminated by well, i.e., well 151 samples differed from well 224 samples irrespectively of MET addition. Bacterial composition in the different microcosms did not converge with time, making it unlikely that storage and incubation had a significant influence.

Overall, obtained data suggest that, even at doses exceeding environmentally relevant and chronic concentrations by one or two orders of magnitude, a 21-day exposure to MET and its degradates modifies the diversity of dominant groups of the bacterial community only to a minor extent. Nevertheless, more subtle changes, e.g., in rare OTUs, may reflect adaptation of bacterial communities to a specific factor such as presence of a micropollutant or its degradation. This type of response, however, may not have to be proportional to exposure dose, and will thus likely be difficult to evidence at environmental concentrations, as shown previously for soil (Crouzet et al., 2010). Worthy of note in the perspective of future studies, temporal trajectories of community changes during incubation may additionally evidence transient responses of groundwater bacterial diversity during MET degradation. The tempo and mode of this response may thus provide additional value of DNA-based information for bioindication of ecosystem status and potential for recovery following toxic impacts.

Further and even if community differentiation between exposure levels can potentially be resolved at the OTU level in some instances, its functional relevance will remain elusive without additional information. In a further attempt to extract useful information, we used Phylogenetic Investigation of Communities by Reconstruction of Unobserved States (PICRUSt, Langille et al., 2013) as a first step toward evaluating functional responses of groundwater bacterial communities exposed to different MET levels.

### Effect of MET Exposure on Predicted Functional Properties of Bacterial Communities

Changes in community functional profiles suggested by PICRUSt were evaluated by cluster analysis, revealing two distinct clusters (**Figure 4B**). As for bacterial community composition, different microcosms were mainly discriminated by groundwater origin. Compared to bacterial composition profiles (**Figure 4A**), however, the relative height of cluster separation was an order of magnitude lower for functional predictions (**Figure 4B**), confirming that changes in minor changes in predicted functions were likely minor.

Interestingly, the three major predicted types of functions in all microcosms were metabolism, genetic information processing and environmental information processing, accounting for 47.1 ± 1.2%, 16.6 ± 0.5%, and 16.3 ± 1.4% (mean ± 1 σ, *n* = 8) of functional counts, respectively (**Supplementary Section K**). Functions for xenobiotics biodegradation and metabolic pathways featured prominently, ranging from 2.2% (experiments with well 224 groundwater exposed to MET) to 2.5% (experiments with well 151 groundwater) of functional counts (**Supplementary Section L**). Predicted pathways for glutathione metabolism, predicted to be associated with chloroacetanilide transformation (Graham et al., 1999) accounted for 0.5% of the functional counts, and did not vary significantly across microcosms (**Supplementary Section L**). In contrast, predicted metabolic pathways for atrazine had very low (≤0.03%) abundances, in agreement with very minor concentrations of atrazine in groundwater samples (**Supplementary Section L**).

### 16S rRNA OTUs as Potential Biomarkers of MET Exposure

PICRUSt-based predictions suggest the occurrence of only slightly distinct gene complements in the bacterial compartment of the two tested groundwater wells, despite markedly different contamination histories. This contrasts with previous work on diverse environments contaminated with hydrocarbons, where specific functional modules were identified that then served as biomarkers to distinguish between different oil contaminated sites (Mukherjee et al., 2017). Thus, and in the absence of known functional markers for MET degradation, we last asked the question whether taxonomical 16S rRNA gene OTUs that exclusively responded to MET as potential biomarkers of MET exposure could be identified, by comparison of identified OTUs across the different microcosms. OTUs which were exclusively found with increasing addition of MET were sought. OTUs potentially correlating with MET exposure varied between 0.4 and 3.6% of total OTUs across all microcosm experiments (**Table 3**). Relative abundance of sequences exclusively found at highest MET amendment (>3.5%) was largest in experiments with well 224 groundwater. In contrast, sequences uniquely found with MET addition in experiments with well 151 represented only 0.4% (0.1 mg L^-1^ MET) and 2.1% (5 mg L^-1^ MET) of total sequences. This suggests that exposure to higher MET levels in groundwater with a history of chronical (well 151) or very low (well 224) exposure to MET was associated with a slight increase of specific taxa. Strikingly, however, OTUs exclusively associated with either unamended or MET-amended conditions were essentially well-specific (**Supplementary Section J**). In other words, generally applicable MET-responding OTUs are likely to be very limited.

**Table 3:** OTUs exclusively found in groundwater experiments with low (no addition) or high exposure to MET (0.1 and 5 mg L^-1^) in wells 151 and 224.

MET exposition	Well	Number of OTUs^a^ (number of sequences)	Relative abundance of specific OTUs [%]^b^							

			151_IGW	151_0	151_0.1	151_5	224_IGW	224_0	224_0.1	224_5
lMET addition (0.1 and 5 mg L^-1^)	151	86 (1671)	0	0	**0.40**	**2.10**	0.18	0.05	0.04	0.05
lNo MET addition (IGW and 0)	151	125 (3913)	**1.00**	**0.73**	0	0	0.20	0.12	1.93	1.89
lMET addition (0.1 and 5 mg L^-1^)	224	172 (5877)	0.17	0.19	0.40	2.50	0	0	**3.60**	**3.50**
lNo MET addition (IGW and 0)	224	43 (4644)	0.12	1.70	2.50	1.61	**0.18**	**1.40**	0	0
l^a^MET addition (0.1 and 5 mg L^-1^)	151 and 224	2 (40)	0	0	**0.02**	**0.1**	0	0	**0.01**	**0.01**
l^a^No MET addition (IGW and 0)	151 and 224	1 (7)	**<0.01**	**0.01**	0	0	**0.01**	**<0.01**	0	0
l

*IGW, initial groundwater. In bold are the relative abundance of sequences of the wells targeted by the search in the given MET exposure conditions.*

*^*a*^OTUs exclusively found in groundwater from both 151 and 224 wells at the given MET exposure conditions.*

*^*b*^Relative abundance of specific OTUs (%) relative to the total number of OTUs found in groundwater microcosms from both 151 and 224 wells at the given MET exposure conditions.*

MET-responding OTUs exclusively found in microcosms with MET addition (0.1 or 5 mg L^-1^) and common to well 151 and well 224 yielded only 2 OTUs (i.e., <0.1% the total number of OTUs) affiliated to *Clostridia* sp., the unclassified OTU00544 and *Fastidiosipila* sp. OTU00881. Conversely, OTU01317 (affiliated to *Gammaproteobacteria)* was the only OTU common to both wells and found exclusively in microcosms to which no MET was added. The association between these two OTUs and MET exposure is still unknown.

While this shows that bacterial OTUs specifically correlated with MET exposure can in principle be identified by high-throughput sequencing, it also confirms that their number will be low, and of uncertain significance in the absence of further information on the corresponding organisms and their metabolic features. In this context, it is worth noting that unclassified bacterial taxa systematically represented over 40% of total sequences. This emphasizes the need to identify and characterize a larger fraction of the bacteria detected in groundwater. Specifically, experiments targeting metabolic activity of identified OTUs of interest (such as OTU00544 and OTU00881 here), e.g., by way of shotgun sequencing of microcosm metagenomes and assembly and analysis of associated contigs (Vanwonterghem et al., 2014), may help to identify organisms and genes potentially involved in MET degradation.

## Conclusion

This study evaluated the response of groundwater bacterial communities exposed to MET and its degradates by coupling *in situ* and *in labo* experiments, including CSIA and a culture-independent 16S rRNA survey. The response of bacterial communities exposed to chloroacetanilide herbicides could not be teased apart from effects of hydrogeochemical conditions and other pesticides *in situ*. Therefore, microcosm investigations on groundwater samples under controlled conditions *in labo* were used to filter out environmental *in situ* variations. Degradation of MET did not correlate with MET exposure level, even at very high MET exposure levels or in groundwater with no records of MET contamination. Moreover, no relationship between bacterial community composition as assessed by 16S rRNA amplicon sequencing and extent of MET degradation was apparent. In other words, detrimental toxic effects of metolachlor on the bacterial compartment of groundwater were not apparent, as also found by the standard Microtox^®^ ecotoxicological assay. Further, functional profiles of bacterial communities as predicted from 16S rRNA by PICRUSt indicated only minor differences in the gene complement of bacterial communities in association with differences in pesticide contamination. Nevertheless, a small number of OTUs whose abundance correlated with levels of MET exposure were identified. This suggests that alterations of groundwater communities exposed to pesticides can be sensitively probed using high-throughput sequencing. In conclusion, this study provides a possible analysis framework to explore the potential of using bacteria as biomarkers of pesticide contamination in aquifers.

## Author Contributions

GI and SV designed the work and critically revised the article. GI, NB, BM, and SD collected and analyzed the samples. GI drafted the article with contributions from LB, NB, and SV. All co-authors analyzed and interpreted the data and approved the final submitted version of the manuscript.

## Conflict of Interest Statement

The authors declare that the research was conducted in the absence of any commercial or financial relationships that could be construed as a potential conflict of interest.

## Footnotes

1 http://trex.biohpc.org

2 http://www.mothur.org/wiki/MiSeq_SOP

3 http://huttenhower.sph.harvard.edu/galaxy/
