# Supplementary material for: Toward Integrative Bacterial Monitoring of Metolachlor Toxicity in Groundwater
Source: Front Microbiol. 2018 Oct 16;9:2053. doi: 10.3389/fmicb.2018.02053 (PMC6198151; doi:10.3389/fmicb.2018.02053)
Supplement: Supplementary file 1 [file Data_Sheet_1.PDF]

Supporting Information

Towards integrative monitoring of metolachlor bacterial toxicity in groundwater

Gwenaël Imfeld[1], Ludovic Besaury[2,3], Bruno Maucourt[2], Stéphanie Donadello[2,4], Nicole Baran[5], Stéphane Vuilleumier[2]*

[1] Université de Strasbourg, EOST-CNRS, LHyGeS UMR 7517, Laboratory of Hydrology and Geochemistry, Strasbourg, France

[2] Université de Strasbourg, CNRS, GMGM UMR 7156, Department of Microbiology, Genomics and the Environment, Strasbourg, France

[3]Université de Reims Champagne Ardenne, INRA, FARE UMR A614, Reims, France (present address)

[4]Soleo Services, Agence Sud Ouest, Lescar, France

[5]BRGM (French Geological Survey), Orléans, France

*Corresponding author phone: e-mail: imfeld@unistra.fr; Phone : + 333 6885 0474

Université de Strasbourg /EOST-CNRS UMR 7517, 1 Rue Blessig, 67000 Strasbourg, France

Manuscript for Frontiers in Microbiology

Table of Contents

Section A. Protocol for carbon stable isotope analysis of MET **3**

Section B. Processing of Illumina Miseq data **4**

Section C. Detailed hydrochemical data of groundwater samples **5**

Section D. Detailed pesticide and TPs concentrations in groundwater **6**

Section E. Detailed hydrochemical data of groundwater MET exposure experiments **8**

Section F. Enumeration of viable microbial cells **9**

Section G. Validation of the Microtox® test for evaluation of MET toxicity **10**

Section H. Rarefaction curves for bacterial OTUs **11**

Section I. Rarefaction curves for Shannon H' and Inverse Simpson diversity **12**

Section J. Relative abundance of specific genera **13**

Section K. Relative abundance of predicted functions **14**

Section L. Relative abundance of potentially relevant metabolisms for MET **15**

Section A. Protocol for carbon stable isotope analysis of MET

The carbon isotope composition of MET in the laboratory exposure experiment was analysed using a GC-C-IRMS system consisting of a TRACE™ Ultra Gas Chromatograph (ThermoFisher Scientific) coupled via a GC IsoLink/Conflow IV interface to an isotope ratio mass spectrometer (DeltaV Plus, ThermoFisher Scientific). The oxidation furnace of the interface was set to a temperature of 1000 °C. A TG-5MS column (30 m × 0.25 mm, 0.25 µm film thickness) was used for chromatographic separation, with helium as the carrier gas at a flow rate of 1.5 mL min^{-1}. The column was held at 50 °C for 1 min, heated at a rate of 15 °C min^{-1} to 150 °C, then up to 250 °C at 2 °C min^{-1}, then heated at 20 °C min-1 to 300 °C and held for 3 min. Samples (3 µL volume) were injected into a split/splitless injector operated in splitless mode and held at 280 °C. The reproducibility of triplicate measurements was ≤ 0.2‰ (1σ). The $\delta^{13}C$ values were calibrated using a three-point calibration against the V-PDB standard using international reference material AIEA600, USGS40 and USGS41 ($\sigma < 0.05$ ‰). The analytical methods were evaluated for possible isotope artifacts since SM water extraction and pre-concentration procedures may alter the measured $\delta^{13}C$ values. The detailed evaluation was described previously in Elsayed et al. (2014) and the same SPE procedure was followed here. The linearity range of the GC–C–IRMS instrument, i.e. the range of injected amount of compound for which the $\delta^{13}C$ values of the compound stays within a ± 0.5 ‰ interval, was established as about 25 to 200 ng of carbon injected on column.

The reproducibility of triplicate measurements was ≤ 0.1‰ (1σ). An in-house chloroacetanilide mix standard with known isotopic composition was measured at least every nine injections to control the quality of measurements. Reference carbon isotope composition values of the pesticide standards were obtained using an elemental analyser-isotopic ratio mass spectrometer coupled to an isotope ratio mass spectrometer (EA IsoLink IRMS system, ThermoFischer Scientific). The carbon isotope ratios were reported in δ notation in parts per thousand [‰] relative to the international carbon isotope standard Vienna Pee Dee Belemnite (V-PDB).

Section B. Processing of Illumina Miseq data

Denoising, chimera checking, generation of operating taxonomic units (OTUs) and taxonomic classification were performed using Mothur software package v.1.33.2 (Schloss et al., 2009) by following the default parameters from the analysis pipeline of Miseq SOP (http://www.mothur.org/wiki/MiSeq_SOP).Briefly, read pairs were assembled into contigs Any contig shorter than (N) and longer than 380 bp were discarded. Unique sequences were screened and were then pre-clustered for up to two differences between sequences. Chimera sequences were checked and removed using UCHIME (Edgar et al., 2011). Sequences were then classified using naïve Bayesian classifier against SILVA bacteria reference database (SSU_Ref database v.102) with boot-strap cut-off of 80%. Sequences related to Bacteria domain were selected and the rest of the sequences was discarded.

To calculate the diversity and richness indices, the Illumina MiSeq sequences were re-analysed using MOTHUR (http://www.mothur.org) starting from denoised and chimera-checked sequences, aligned, and clustered to define OTUs at 98% sequence identity. A subsample of sequences was then randomly selected to obtain equally sized datasets according to the standard operating procedure (Schloss et al. 2009). The resulting datasets were used to calculate the diversity indices using R and for rarefaction analysis. Shannon's diversity index (H') was calculated as $H' = -\sum p_i \ln p_i$, and the inverse Simpson's diversity index (S) was calculated as $S = 1/(1 - D)$ with $D = \sum p_i^2$, where p_i is the relative abundance of species i. The Chao1 richness estimate was calculated as $S_{chao1} = S_{obs} + {f_1}^2/2f_2$, where S_{obs} is total number of OTUs in a sample, f_1 is the number of OTUs with only one sequence (i.e., "singletons") and f_2 is the number of OTUs with only two sequences (i.e., "doubletons").

Section C. Hydrogeochemical data of groundwater samples collected in Ariège alluvial plain (France) on May (C1), July (C2), September (C3) and December (C4) 2012, and February (C5) and May (C6) 2013. n.a: not available.

	**MET**	**MET OXA**	**MET ESA**	**EC**	**pH**	**O_2**	**Eh**	**Ca^{2+}**	**Cl^-**	**K^+**	**Mg^{2+}**	**NO_3^-**	**Na^+**	**SO_4^{2-}**	**HCO_3^-**
	**[µg L^{-1}]**	**[µg L^{-1}]**	**[µg L^{-1}]**	**[µS cm^{-1}]**	**[-]**	**[mg L^{-1}]**	**[mV]**	**[mg L^{-1}]**	**[mg L^{-1}]**	**[mg L^{-1}]**	**[mg L^{-1}]**	**[mg L^{-1}]**	**[mg L^{-1}]**	**[mg L^{-1}]**	**[mg L^{-1}]**
151C1	12.2	2.954	3.891	724	6.92	6.72	278	138.7	26.8	2.4	4.2	42.8	23.8	28.9	375
60C1	0.082	0.033	0.11	517	6.77	7.11	145	88.3	21	2.6	5	45.9	9.5	23.2	202
117C1	0.024	0	1.841	405	6.49	4.50	101	34.1	27.4	0.8	13.2	37.3	25.6	27.8	112
230C1	0	0	0.995	491	7.01	5.09	100	94.4	14.6	0.7	2.5	24.9	8.8	15.1	244
224C1	0	0	1.281	399	6.17	5.83	114	37.3	29.2	1	10.4	85.4	22.9	15.3	67
151C2	3.07	1.072	1.401	787	7.06	5.31	266	133.3	28.9	2.3	4	38.8	25.1	31.6	385
60C2	0.401	0.031	0.194	551	7.02	8.43	372	82.4	20	2.5	4.6	45.4	9.9	22.9	194
117C2	0.264	0.01	2.714	434	6.53	2.67	252	32.1	29.1	0.9	12.5	34.4	26.6	29	120
230C2	0	0	0.795	551	7.02	8.43	372	96.3	14.4	0.5	2.5	29.8	9.1	16.4	261
224C2	0	0	1.094	427	6.33	4.58	312	34.4	29.4	0.9	9.4	86.5	21.9	15.6	71
151C3	0.93	0.506	0.75	758	6.59	4.47	203	132.1	29.9	2.5	4.1	33.8	26	32.9	384
230C3	0	0	1.244	525	6.63	3.97	256	100.1	15.9	0.6	3	35.7	10.5	18.9	263
151C4	2.963	1.784	2.623	775	7.21	6.53	265	132.1	30.4	2.4	4	38.9	27.6	29.8	390
60C4	0.043	0.02	0.104	542	6.92	6.44	320	86.3	22.9	3	4.9	52.8	10	22.6	216
117C4	0.071	0.01	3.105	386	6.84	6.30	317	30.4	29.7	0.8	11.6	21	26.5	23.8	130
230C4	0	0	1.337	554	7.38	9.89	326	100.2	17	0	2.6	32.7	9.3	22.9	273
224C4	0	0	1.518	408	6.33	5.62	328	35.5	32.6	1	9.6	89.9	22.7	11.5	73
151C5	4.91	2.7	3.89	678	7.35	8.31	262	107.5	28.8	6.5	4.5	60.1	22	28.8	280
60C5	4.25	4.86	3.24	602	7.08	8.35	352	90.6	24.9	3.1	5.1	64.3	9.9	25.6	197
117C5	n.a.	n.a.	n.a.	847	6.68	7.16	207.3	100	46.4	7.5	19	188.9	31.4	59.6	169
230C5	0.005	0.47	3.19	547	7.10	9.33	340	90.7	25	0	2.6	25.3	12.1	23.6	232
224C5	0	0	1.07	414	6.58	6.23	347	35.3	30.4	1	9	82.6	20.7	16.2	73
151C6	6.392	1.98	2.25	770	7.26	5.28	228	126.1	31.2	4.6	4.3	52.4	19.8	32.8	343
60C6	2.101	1.15	1.72	657	7.03	6.28	288	105.5	29.4	3.8	5.9	102.1	10.9	34.3	219
117C6	0.13	0.145	3.73	599	6.63	0.66	306	49.3	41.4	1.5	18.7	143.5	30.8	29.5	112
230C6	0.067	0.078	1.3	621	7.12	8.34	348	111.1	21.8	0.6	3.2	49.3	10.7	26.9	283
224C6	0	0	0.877	420	6.33	5.02	336	36.3	34.6	1	10	101	22.9	19	75

Section D. Pesticide and TPs concentrations [µg.L^{-1}] in groundwater from well 151 (historically contaminated) and 224 (historically not contaminated) of the Ariège alluvial plain (France) on May (C1), July (C2), September (C3) and December (C4) 2012, and February (C5) and May (C6) 2013.

	**Well 151**	***Metolachlor***	OXA metolachlor	ESA metolachlor	Acetochlor	OXA acetochlor	ESA acetochlor	Alachlor	OXA alachlor	ESA alachlor	Simazine	Flusilazole	Tebuconazole	Atrazine	Desethylatrazine	Chlortoluron	Linuron	Metazachlor	Desmetryn
151	C1	***12.20***	2.95	3.89	0.01	0	0	0	0	0	0	0.01	0.04	0	0.06	0	0.01	0	0.41
	C2	***3.07***	1.07	1.40	0	0	0	0	0	0	0	0	0.01	0	0.05	0	0	0	0.11
	C3	***0.93***	0.51	0.75	0	0	0	0	0	0	0	0	0	0	0.05	0	0	0	0.04
	C4	***2.96***	1.78	2.62	0	0	0	0	0	0	0	0	0.01	0	0.05	0	0	0	0.18
	C5	***4.91***	2.70	3.89	0	0	0	0	0.06	0.12	0	0	0.07	0	0.02	0.25	0	0	0.32
	C6	***6.39***	1.98	2.25	0	0	0	0.01	0.02	0.06	0	0.01	0.06	0	0.03	0.01	0	0	0.15
	Mean	***5.08***	*1.83*	*2.47*	*0*	*0*	*0*	*0*	*0.01*	*0.03*	*0*	*0*	*0.03*	*0*	*0.04*	*0.04*	*0*	*0*	*0.20*
	2σ	***3.95***	*0.94*	*1.28*	*0*	*0*	*0*	*0*	*0.02*	*0.05*	*0*	*0.01*	*0.03*	*0*	*0.02*	*0.10*	*0*	*0*	*0.14*

	**Well 224**	***Metolachlor***	OXA metolachlor	ESA metolachlor	Acetochlor	OXA acetochlor	ESA acetochlor	Alachlor	OXA alachlor	ESA alachlor	Simazine	Flusilazol	Tebuconazol	Atrazine	Desethylatrazine	Chlortoluron	Linuron	Metazachlor	Desmetryn
224	C1	***0***	0	1.28	0	0	0	0	0	0.07	0.04	0	0	0.08	0.46	0	0	0	0
	C2	***0***	0	1.09	0	0	0	0	0	0.05	0.04	0	0	0.08	0.48	0	0	0	0
	C3	***0***	0	1.66	0	0	0	0	0	0.08	0.03	0	0	0.08	0.50	0	0	0	0
	C4	***0***	0	1.52	0	0	0	0	0	0.08	0.04	0	0	0.09	0.36	0	0	0	0
	C5	***0***	0	1.07	0	0	0	0	0	0.03	0.04	0	0	0.12	0.46	0	0	0	0
	C6	***0***	0	0.88	0	0	0	0	0	0.09	0.04	0	0	0.09	0.39	0	0	0	0
	Mean	***0***	*0*	*1.25*	*0*	*0*	*0*	*0*	*0*	*0.07*	*0.04*	*0*	*0*	*0.09*	*0.44*	*0*	*0*	*0*	*0*
	2σ	***0***	*0*	*0.29*	*0*	*0*	*0*	*0*	*0*	*0.02*	*0*	*0*	*0*	*0.02*	*0.05*	*0*	*0*	*0*	*0*

Section E. Hydrochemical data of groundwater MET exposure experiments from well 151 (historically contaminated) and 224 (historically not contaminated) of the Ariège alluvial plain (France) after 21 days of incubation.

Well	Exposure to MET [mg.L^{-1}]	pH	EC [µS.cm^{-1}]	[a]NH_4^+	[a]Na^+	[a]K^+	[a]Mg^{2+}	[a]Ca^{2+}	[a]Inorg. C	[a]Cl^-	[a]NO_3^-	[a]SO_4^{2-}	[a]PO_4^{3-}	DOC [ppm C]
151	No MET addition	8-9	430	<LOD	0.66	0.10	0.15	2.26	45.4	0.56	0.38	0.25	<LOD	n.a.
	0.1	8-9	500	<LOD	0.88	0.13	0.20	2.42	48.1	0.77	0.61	0.32	<LOD	113.4
	5	8-9	600	<LOD	0.90	0.13	0.21	2.53	62.7	0.77	0.71	0.33	<LOD	n.a.
224	No MET addition	8-9	380	0.15	1.05	0.04	0.42	1.09	17.5	0.93	1.36	0.18	0.11	6.7
	0.1	8-9	280	<LOD	0.87	0.04	0.35	0.75	12.4	0.77	1.03	0.13	<LOD	40.4
	5	8-9	350	<LOD	1.01	0.04	0.42	1.15	15.3	0.89	1.26	0.16	0.22	76.0

[a][mmol L^{-1}]

Section F. Enumeration of viable microbial cells in the laboratory exposure experiments by R2A agar plate counts (incubation: 20°C, 48 h).

Well	Exposure to MET [mg.L^{-1}]	Number of viable cells [CFU.mL^{-1}]
151	Initial groundwater	4.8×10^3
	0 (no MET addition)	4.4×10^3
	0.1	6.6×10^3
	5	5.5×10^3
224	Initial groundwater	7.4×10^2
	0 (no MET addition)	7.4×10^2
	0.1	3.0×10^3
	5	2.5×10^3

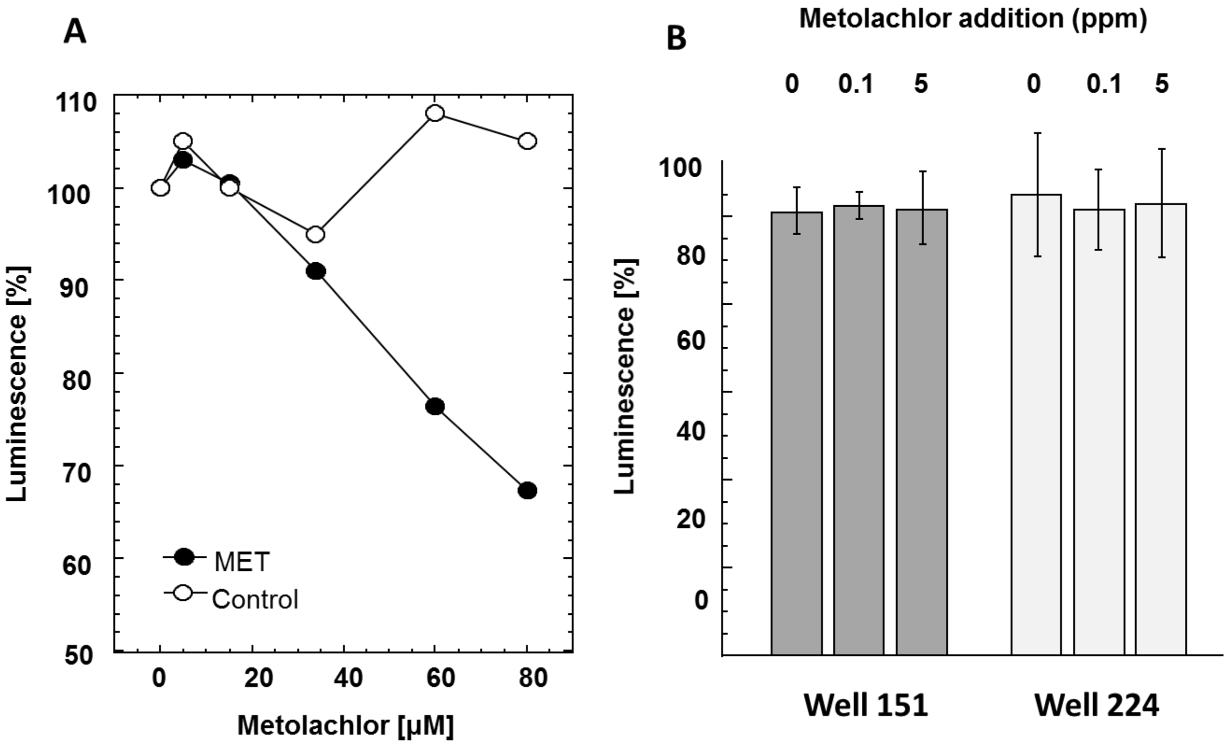

Section G. Validation of the Microtox® test for MET toxicity in pure water (A) and microcosm spent medium (B). The results show an EC_{20} for MET higher than 40 µM (i.e., 11.35 mg.L^{-1}), which predicts the absence of detectable toxicity for MET in exposure experiment with maximum of 5 mg L^{-1}. No effect was observed for groundwater samples, confirming that potential toxic effects of MET as well as of MET degradates could not be detected using the Microtox® assay at the maximal MET dosage used in microcosm experiments. Relative errors in luminescence values [%] are below 10%.

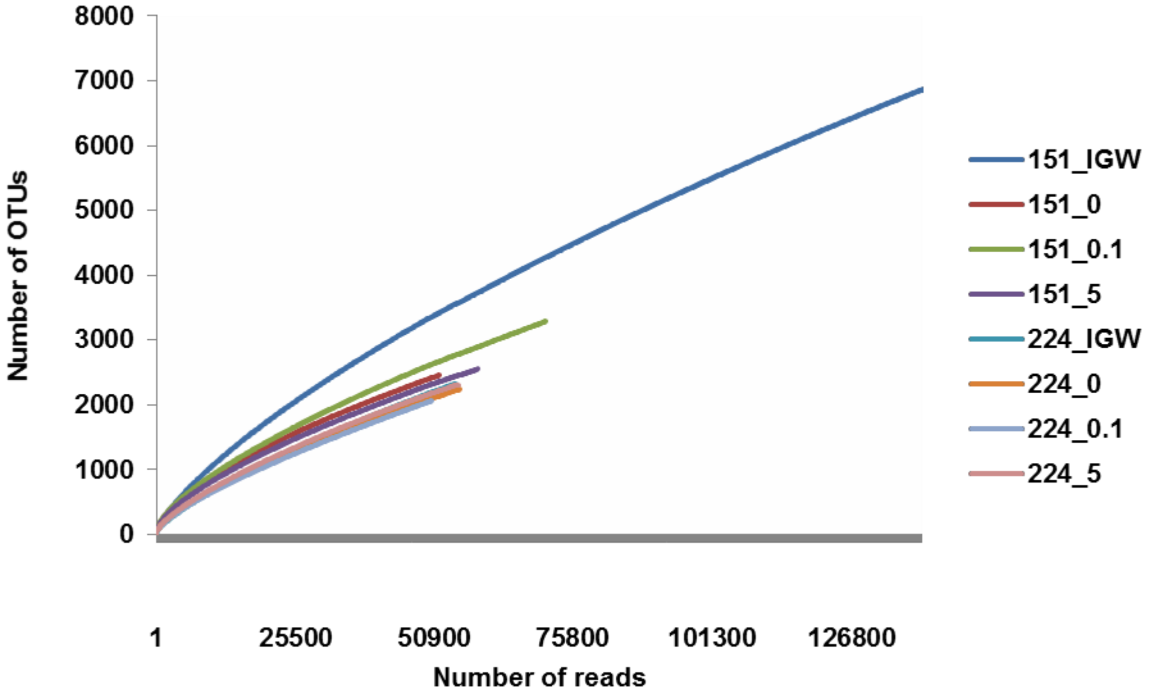

Section H. Rarefaction curves for bacterial OTUs clustering at 98% sequence identity. Curves are shown for groundwater from wells 151 and 224 (IGW: initial groundwater) without MET addition (0) or exposed to 0.1 mg.L^{-1} (_0.1) and 5 mg.L^{-1} (_5) MET during 21 days at 20°C in laboratory exposure experiments.

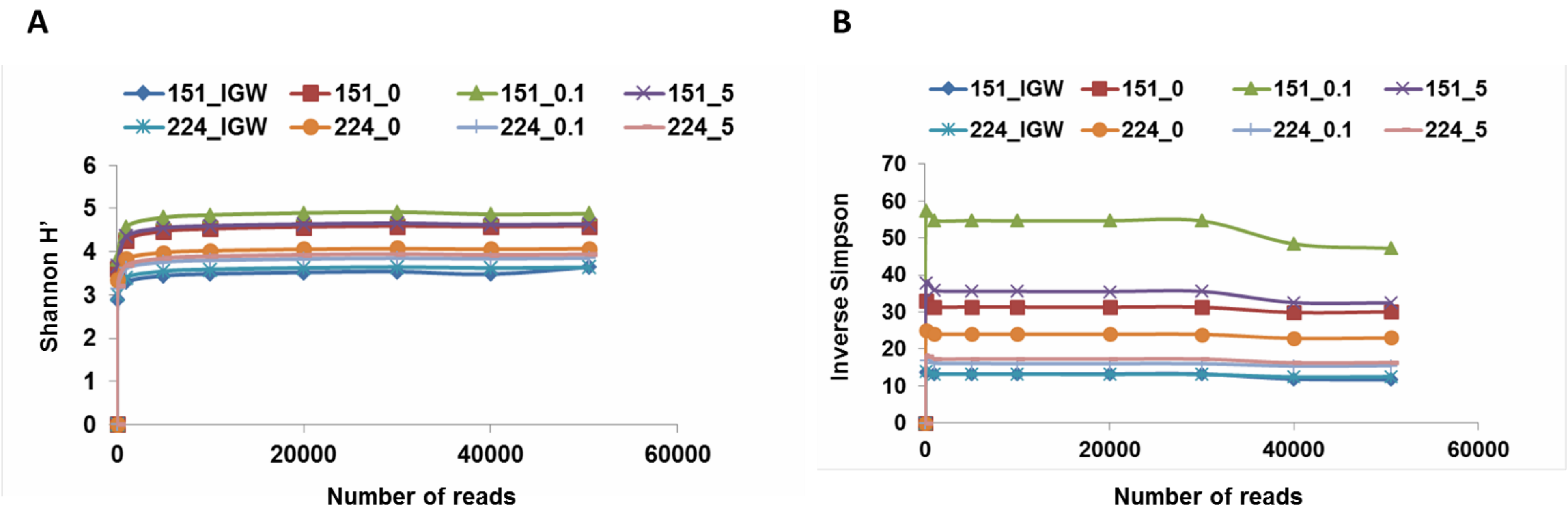

Section I. Shannon H' (A) and Inverse Simpson (B) diversity (98% sequence identity) as a function of number of reads. Curves are shown for groundwater from wells 151 and 224 without MET addition (0) or exposed to 0.1 mg L^{-1} and 5 mg L^{-1} MET during 21 days at 20°C in laboratory exposure experiments. IGW: initial groundwater.

Section J. Relative abundance [%] of genera (98% sequence identity clustering) exclusively found in experiments with low (no addition) or high exposure to MET in wells 151 and 224 without MET addition (0) or exposed to 0.1 mg L^{-1} and 5 mg L^{-1} MET. IGW: initial groundwater.

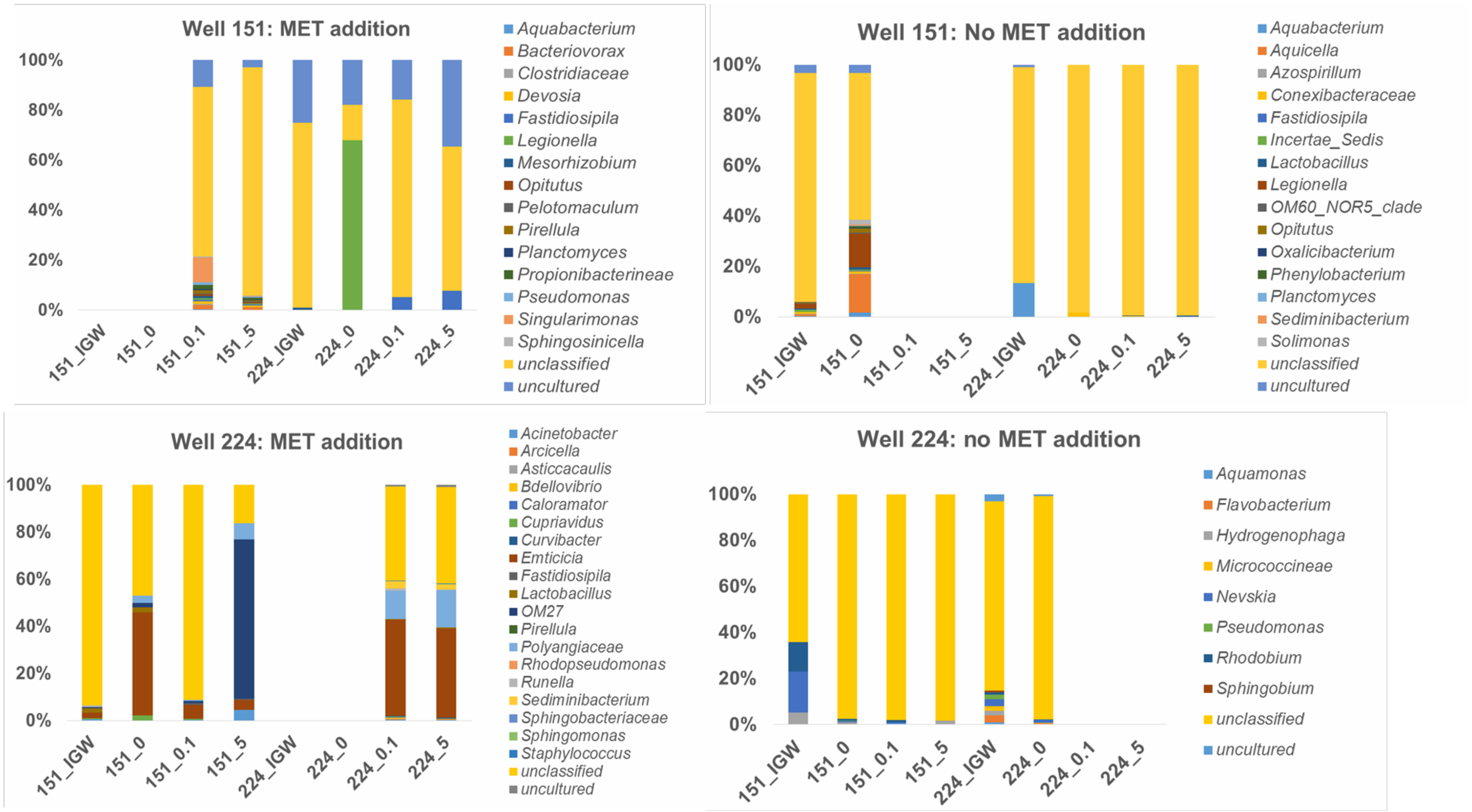

Section K. Relative abundance [% functional counts] of predicted functions from 16S rRNA gene analysis of groundwater bacterial microbiome using PICRUSt analysis for wells 151 and 224 without MET addition (0) or exposed to 0.1 mg L^{-1} and 5 mg L^{-1} MET. IGW: initial groundwater.

Predicted function[a]	151_IGW	151_0	151_0.1	151_5	224_ IGW	224_0	224_0.1	224_5	**Mean[b]**	**1σ**
Metabolism	47.6	48.1	48.0	48.3	45.1	47.9	45.9	46.1	**47.1**	**1.2**
Genetic Information Processing	16.8	17.1	17.1	16.7	15.6	16.9	16.2	16.3	**16.6**	**0.5**
Environmental Information Processing	15.3	15.2	15.2	15.4	18.6	15.1	17.8	17.6	**16.3**	**1.4**
Cellular Processes	4.1	3.7	3.8	3.5	4.4	3.8	3.8	3.8	**3.9**	**0.3**
Human Diseases	1.0	0.9	0.9	0.9	0.9	0.9	0.9	0.9	**0.9**	**0.0**
Organismal Systems	0.7	0.7	0.7	0.7	0.6	0.7	0.6	0.6	**0.7**	**0.1**
Unclassified	14.5	14.2	14.3	14.4	14.7	14.7	14.7	14.7	**14.5**	**0.2**

[a]KEGG pathway and orthology terms

[b]mean ± 1σ of the 8 samples

Section L. Relative abundance [% functional counts] of potentially relevant metabolisms for MET predicted from 16S rRNA gene analysis of groundwater bacterial microbiome using PICRUSt analysis for wells 151 and 224 without MET addition (0) or exposed to 0.1 mg L^{-1} and 5 mg L^{-1} MET. IGW: initial groundwater.

Level	Predicted function[a]	151_IGW	151_0	151_0.1	151_5	224_IGW	224_0	224_0.1	224_5
1	Metabolism	49.4	49.8	49.6	50.0	47.1	49.6	47.7	48.0
2	Xenobiotics Biodegradation and Metabolism	2.5	2.5	2.3	2.5	2.3	2.4	2.2	2.2
3	Atrazine degradation	0.02	0.02	0.02	0.02	0.02	0.02	0.03	0.03
3	DDT degradation	<0.01	<0.01	<0.01	<0.01	<0.01	<0.01	<0.01	<0.01
2	Metabolism of other amino acids	1.6	1.6	1.6	1.6	1.6	1.6	1.6	1.6
3	Glutathione metabolism	0.5	0.5	0.5	0.5	0.5	0.5	0.5	0.5

[a]KEGG pathway and orthology terms
